# Examining Primary Care Utilization and Distance Traveled for Care Among Medicaid-Insured Children in South Carolina

**DOI:** 10.1007/s10900-025-01492-4

**Published:** 2025-06-10

**Authors:** Caitlin Koob, Abass Babatunde, Samuel L. K. Baxter, Michelle Parisi, Sarah F. Griffin

**Affiliations:** 1 Department of Healthcare Leadership and Management, Medical University of South Carolina, Charleston, SC USA; 2 Department of Public Health Sciences, Clemson University, Clemson, SC USA; 3 Department of Health Policy and Management, University of North Carolina, Chapel Hill, NC USA; 4 Department of Nutritional Sciences, University of Georgia, Athens, GA USA

**Keywords:** Pediatric obesity, Obesity prevention and treatment, Primary care, Healthcare utilization, Rural health

## Abstract

To identify demographic and primary care utilization factors associated with distance traveled for healthcare among Medicaid-insured children in South Carolina (SC). Medicaid claims data was analyzed among patients with a weight-related diagnostic code who received primary care services in SC from 2018 to 2022 (*N* = 1,055,613). Multinomial regression analyses were used to examine associations between demographic and primary care utilization factors and distance traveled to primary care services. Among Medicaid-insured children in SC, those who were Non-Hispanic White, obese, had lapses in coverage, sought non-well-child visits, and lived in rural counties demonstrated higher odds of traveling for primary care, compared to other children. Further, children who were Non-Hispanic White, overweight, and had lapses in coverage demonstrated higher odds of traveling distally for PC, compared to other children. These findings underscore the complex interplay between race/ethnicity, healthcare access, and obesity risk among Medicaid-insured children, emphasizing the need for targeted interventions that address these disparities. The study’s insights can inform policy and healthcare practices aimed at improving the accessibility of primary care and supporting effective weight management strategies for pediatric populations in SC.

## Introduction

 In South Carolina (SC), 21.6% of children aged 10–17 are affected by obesity, with the seventh-highest statewide prevalence relative to the national statistics [1–3]. The COVID-19 pandemic, causing increased stress, sedentary behaviors, and social isolation, exacerbated pediatric obesity rates nationwide [4]. Over time, children diagnosed as overweight and obese are at-risk of developing mental health conditions, sleep disturbances, and chronic diseases across the lifespan [2–6]. SC serves as a case study for the Southern region of the U.S., where lifestyle behaviors, healthcare infrastructure, and policies influence long-term health [6–9]. Previous research found improved primary care (PC) access along highway systems in SC, highlighting the context of place in rural health among pediatrics [10]. Additional research is needed to identify multi-level interventions that support healthy weight management among children in rural communities, in an effort to reduce their risks of chronic disease and associated comorbidities into adulthood [2, 4, 7]. 

According to the American Academy of Pediatrics’ (AAP) clinical practice guidelines, the social-ecological context of pediatric obesity *must* be considered during the assessment, prevention, and/or treatment phases of PC [11]. These guidelines promote a multidisciplinary approach to pediatric weight management, recommending frequent follow-up visits, family-level lifestyle behavior changes, and reinforcement for targeted behavior change beyond weight-specific interventions [11]. Still, children with Medicaid insurance, gaps in insurance coverage, in low-income households, and who are boys and/or non-Hispanic Black demonstrate limited healthcare access and less likelihood of participating in pediatric weight management programs through PC clinics [12–15]. Previous studies examined the associations between patient-, household-, and community-level factors and weight classification [15, 16]; however, little research examines where Medicaid-insured children are receiving PC for weight management in the South, where pediatric obesity rates are disproportionately higher than national rates (36.6% vs. 30.7%) [17].

Still, the demographic makeup of SC is comparable to other Southern states; however, SC has historically opposed Medicaid expansion and is considered low-income with a median household income of $43,290 [18–20]. Further, with 35 healthcare facilities per 100,000 residents, previous research found the highest healthcare infrastructure density among two most populous cities in SC, and significantly less in surrounding counties [18]. Therefore, this study aims to identify demographic and PC utilization factors associated with distance traveled for care among Medicaid-insured children in SC.

## Methods

### Study Design

This study employed a retrospective, observational design utilizing a dataset of PC visits among Medicaid-insured children in SC, sourced from SC Integrated Data System housed at the SC Revenue and Fiscal Affairs Office. Patients are represented cross-sectionally at most recent PC visit. Further, this study has been approved by Clemson University’s Institutional Review Board.

### Data Collection

This data is sourced from 2018 to 2022 and includes visit-related variables, such as visit type and weight-related ICD-10 code(s) [6, 21], geographic variables (county of service and patient residence), and demographic variables.

Through the data cleaning process, claims indicating “unknown race/ethnicity” (*n* = 1,311,537) were excluded. Additionally, those who reportedly received services in McCormick County but did not live in McCormick or an adjacent county (*n* = 534,037) were excluded. McCormick County houses a billing processing center, which inaccurately indicated patients traveled far distances for PC in this county. Lastly, this dataset is restricted to PC visits. Demographics were compared between the sample population and the full dataset at each step and remained analogous. In total, approximately 66.94% of the original dataset was lost. Still, the sample maintains adequate power for this analysis. The final dataset includes 1,055,613 observations of Medicaid-insured children with a BMI-related ICD-10 code from 2018 to 2022.

### Outcome Variable

The primary outcomes in this study were distance traveled for PC, measured by distance from patient’s county of residence to service county. The categorical variable for distance traveled for care had three levels for PC services provided: (1) local, (2) proximal, or (3) distal. Those who received PC within their county of residence were described as receiving “local” care. Patients who traveled to an adjacent county for PC were described as receiving “proximal” care. Children who traveled beyond their adjacent counties for PC received “distal” care. Boundaries for “proximal” and “distal” care were established based on the SC Department of Transportation guidelines [22]. 

### Primary Care Utilization

Independent variables include patients’ body mass index (BMI) classification, lapses in Medicaid coverage (continuous), visit type (well-child [WCV] or non-well-child visit [non-WCV]), and rurality (rural/non-rural) [23]. BMI classification was categorized into three groups: (1) “healthy weight” (less than 85th percentile), (2) “overweight” (85th to 95th percentile), and (3) “obesity” (greater than the 95th percentile) based on ICD-10 codes [11, 21]. Rural classification was determined based on previous pediatric obesity research in SC [10, 23]. Continuous Medicaid enrollment, accessible routine and preventative care, and rurality have been previously identified as predictors of PC utilization and weight-related concerns, justifying their inclusion [10, 19, 24–26]. 

### Demographic Variables

Covariates include demographic variables, such as age (in years), race/ethnicity (Non-Hispanic White, Non-Hispanic Black, Hispanic, Other), and biological sex (boys/girls).

### Statistical Analysis

Descriptive statistics, such as chi-square tests, were used to examine patient characteristics and their association(s) with distance traveled for PC (local, proximal, distal) among the sample. Multinomial logistic regression models were fit to examine: (1) associations between the predictor variables and the distance traveled to PC, and (2) differences in PC utilization by BMI classification. Multinomial logistic regression is well-suited for analyzing multi-level categorical outcome variables [27]. This analysis used the *multinom* function from the “nnet” package in R [27]. 

## Results

The mean age of patients was 10.8 years (*SD* = 4.04) and half were girls (50.2%; Table 1). Most patients were diagnosed with a healthy weight (58.1%), received non-WCV (81.1%), with continuous coverage (77.7%), and lived in non-rural counties (79.8%). The majority of patients were Non-Hispanic White (42.6%) or Non-Hispanic Black (46.4%).

**Table 1:** Characteristics of Medicaid-insured children with receipt of primary care services in South Carolina, 2018–2022 (*N* = 1,055,613)

	Overall (*N* = 1,055,613)	Distance traveled for care			*p*-value
		Local care **(*n*** **= 694**,**866)**	Proximal care **(*n*** **= 209**,**355)**	Distal care **(*n =*** **151**,**392)**	
Age, *M* (SD)					**< 0.001**
Mean (SD)	10.8 (4.04)	10.9 (4.01)	10.7 (4.12)	10.9 (4.02)	
Median [Min, Max]	11.0 [0, 18.0]	11.0 [0, 18.0]	11.0 [0, 18.0]	11.0 [0, 18.0]	
Sex, ***n*** (%)					0.745
Girls	530,202 (50.2)	348,826 (50.2)	105,239 (50.3)	76,137 (50.3)	
Boys	525,411 (49.8)	346,040 (49.8)	104,116 (49.7)	75,255 (49.7)	
Race and/or Ethnicity, ***n*** (%)					**< 0.001**
Non-Hispanic White	449,792 (42.6)	285,199 (41.0)	99,490 (47.5)	65,103 (43.0)	
Non-Hispanic Black	489,907 (46.4)	327,294 (47.1)	91,294 (43.6)	71,319 (47.1)	
Hispanic	82,925 (7.9)	60,364 (8.7)	11,741 (5.6)	10,820 (7.1)	
Other	32,989 (3.1)	22,009 (3.2)	6830 (3.3)	4150 (2.7)	
BMI obesity classification, ***n*** (%)					**< 0.001**
5th-85th Percentile	613,035 (58.1)	404,860 (58.3)	122,772 (58.6)	85,403 (56.4)	
85th-95th Percentile	327,585 (31.0)	213,942 (30.8)	62,527 (29.9)	51,116 (33.8)	
> 95th Percentile	114,993 (10.9)	76,064 (10.9)	24,056 (11.5)	14,873 (9.8)	
Number of lapses in coverage per patient, ***n*** (%)					**< 0.001**
0	820,404 (77.7)	549,736 (79.1)	155,403 (74.2)	115,265 (76.1)	
1	12,408 (1.2)	7250 (1.0)	3141 (1.5)	2017 (1.3)	
2	25,874 (2.5)	15,517 (2.2)	6173 (2.9)	4184 (2.8)	
3	36,660 (3.5)	22,201 (3.2)	8402 (4.0)	6057 (4.0)	
4	48,044 (4.6)	30,091 (4.3)	10,731 (5.1)	7222 (4.8)	
5	51,725 (4.9)	32,247 (4.6)	11,688 (5.6)	7790 (5.1)	
6	60,498 (5.7)	37,824 (5.4)	13,817 (6.6)	8857 (5.9)	
Visit type, ***n*** (%)					
Non well-child visit	124,730 (81.1)	562,386 (80.9)	168,680 (80.6)	124,730 (82.4)	
Well-child visit	199,817 (18.9)	132,480 (19.1)	40,675 (19.4)	26,662 (17.6)	
Rurality, ***n*** (%)					**< 0.001**
Rural	213,004 (20.2)	102,045 (14.7)	51,246 (24.5)	59,713 (39.4)	
Non-rural	842,609 (79.8)	592,821 (85.3)	158,109 (75.5)	91,679 (60.6)	

Bold values indicate statistical significance (*p* < 0.001)

Further, the sample demonstrated no significant differences in sex or distance traveled for PC (*p* = 0.745). However, a significantly higher proportion of Non-Hispanic Black patients received care locally or traveled distally for PC, compared to other racial and/or ethnic groups (*p* < 0.001). Furthermore, children diagnosed as overweight and obese were significantly less likely to travel for PC, compared to children with healthy weight (*p* < 0.001). In addition, lapses in Medicaid coverage by patient, visit type, and rurality significantly differed across distance traveled for PC (*p* < 0.001 for each).

### Proximal Care

Table 2 demonstrates significant differences in patient-level variables associated with distance traveled for PC. For children receiving proximal PC, age showed a significant negative association (aOR = 0.99, 95% CI: [0.99, 0.99], *p* < 0.001) with each year compared to children receiving local PC. Relative to non-Hispanic White children, non-Hispanic Black children had 23% lower odds (aOR = 0.77, 95% CI: [0.77, 0.78], *p* < 0.001) and Hispanic children had 47% lower odds (aOR = 0.53, 95% CI: [0.52, 0.54], *p* < 0.001) of traveling to receive proximal PC versus local PC. Children of other racial and/or ethnic groups also had 22% lower odds of receiving proximal PC versus local PC (aOR = 0.88, 95% CI: [0.86, 0.91], *p* < 0.001), compared to non-Hispanic White children. Pediatric weight classification demonstrated a significant positive association, where children diagnosed as obese had 12% higher odds of receiving proximal PC versus local PC (aOR = 1.12, 95% CI: [1.10, 1.13], *p* < 0.001), compared to children diagnosed as healthy weight. There was no significant difference between sex or children classified as overweight versus healthy weight (*p* = 0.756) in distance traveled for PC.

**Table 2:** Multinomial regression analysis: strength and significance of effects on distance to receipt of primary care among Medicaid-insured children in South Carolina, 2018–2022 (*N* = 1,055,613)

	Proximal (vs. Local)			Distal (vs. Local)		
	Adjusted Odds Ratio	95% CI	*p*	Adjusted Odds Ratio	95% CI	*p*
Age	0.99	[0.99, 0.99]	**< 0.001**	1.00	[1.00, 1.00]	0.483
Race (Ref: Non-Hispanic White)						
Non-Hispanic Black	**0.77**	**[0.77**,** 0.78]**	**< 0.001**	**0.87**	**[0.86**,** 0.88]**	**< 0.001**
Hispanic	**0.53**	**[0.52**,** 0.54]**	**< 0.001**	**0.67**	**[0.65**,** 0.68]**	**< 0.001**
Other	**0.88**	**[0.86**,** 0.91]**	**< 0.001**	**0.85**	**[0.82**,** 0.88]**	**< 0.001**
Sex (Ref: Girls)						
Boys	1.00	[0.99, 1.01]	0.467	1.01	[1.00, 1.02]	0.095
BMI Obesity Classification (Ref: 5th-85th Percentile)						
85th-95th Percentile	1.00	[0.99, 1.01]	0.595	**1.13**	**[1.11**,** 1.14]**	**< 0.001**
> 95th Percentile	**1.12**	**[1.10**,** 1.13]**	**< 0.001**	**0.97**	**[0.95**,** 0.99]**	**0.004**
Number of lapses in coverage per patient (Ref: 0)						
** 1**	**1.53**	**[1.47**,** 1.60]**	**< 0.001**	**1.40**	**[1.33**,** 1.48]**	**< 0.001**
** 2**	**1.39**	**[1.35**,** 1.44]**	**< 0.001**	**1.33**	**[1.29**,** 1.38]**	**< 0.001**
** 3**	**1.33**	**[1.30**,** 1.37]**	**< 0.001**	**1.33**	**[1.29**,** 1.37]**	**< 0.001**
** 4**	**1.26**	**[1.23**,** 1.28]**	**< 0.001**	**1.17**	**[1.14**,** 1.20]**	**< 0.001**
** 5**	**1.27**	**[1.25**,** 1.30]**	**< 0.001**	**1.17**	**[1.14**,** 1.20]**	**< 0.001**
** 6**	**1.29**	**[1.26**,** 1.31]**	**< 0.001**	**1.13**	**[1.10**,** 1.16]**	**< 0.001**
Visit Service Type (Ref: Well-Child Visit)						
Non well-child visit	**1.02**	**[1.01**,** 1.04]**	**< 0.001**	**0.91**	**[0.90**,** 0.92]**	**< 0.001**
Rurality (Ref: Non-rural)						
Rural	**1.96**	**[1.94**,** 1.99]**	**< 0.001**	**0.26**	**[0.26**,** 0.26]**	**< 0.001**

Bold values indicate statistical significance (*p* < 0.001)

Compared to children who did not experience a lapse in Medicaid coverage, children with one lapse in coverage had 53% higher odds of receiving proximal PC versus local PC (aOR = 1.53, 95% CI: [1.47, 1.60], *p* < 0.001). Children with two to six lapses in coverage had 26–39% higher odds of receiving proximal PC versus local PC (2 lapses: aOR = 1.39, 95% CI: [1.35, 1.44], *p* < 0.001; 3 lapses: aOR = 1.33, 95% CI: [1.30, 1.37], *p* < 0.001; 4 lapses: aOR = 1.26, 95% CI: [1.23, 1.28], *p* < 0.001; 5 lapses: aOR = 1.27, 95% CI: [1.25, 1.30], *p* < 0.001; 6 lapses: aOR = 1.29, 95% CI: [1.26, 1.31], *p* < 0.001), compared to children with no lapse.

Further, children seen for non-WCV had slightly higher odds of traveling for proximal PC versus local PC (aOR = 1.02, 95% CI: [1.01, 1.04], *p* < 0.001), compared to children seen for WCV. Children in rural areas had 96% higher odds of traveling for proximal PC versus local PC (aOR = 1.96, 95% CI: [1.94, 1.99], *p* < 0.001), compared to children in non-rural areas. There was no significant association with sex and receiving proximal PC versus local PC (*p* = 0.467).

### Distal Care

Relative to Non-Hispanic White children, Non-Hispanic Black children had 13% lower odds (aOR = 0.87, 95% CI: [0.86, 0.88], *p* < 0.001) and Hispanic children had 43% lower odds (aOR = 0.67, 95% CI: [0.65, 0.68], *p* < 0.001) of receiving distal PC versus local PC. Additionally, children of other racial/ethnic groups had 15% lower odds (aOR = 0.85, 95% CI: [0.82, 0.88], *p* < 0.001) of receiving distal PC versus local PC, compared to Non-Hispanic White children.

Compared to children with healthy weight, children diagnosed as overweight had 13% higher odds of receiving distal PC versus local PC (aOR = 1.13, 95% CI: [1.11, 1.14], *p* < 0.001). Conversely, children diagnosed as obese had 3% lower odds (aOR = 0.97, 95% CI: [0.95, 0.99], *p* = 0.004) of receiving distal PC versus local PC, compared to children with healthy weight.

The number of lapses in Medicaid coverage per patient was positively associated with receiving distal PC versus local PC, compared to children without a lapse in coverage (*p* < 0.001). Children with one lapse in coverage demonstrated 40% higher odds of receiving distal PC versus local PC (aOR = 1.40, 95% CI: [1.33, 1.48], *p* < 0.001), compared to children with no lapse. Additionally, children with two to six lapses in coverage had 13–33% higher odds of receiving distal PC versus local PC (2 lapses: aOR = 1.33, 95% CI: [1.29, 1.38], *p* < 0.001; 3 lapses: aOR = 1.33, 95% CI: [1.29, 1.37], *p* < 0.001; 4 lapses: aOR = 1.17, 95% CI: [1.14, 1.20], *p* < 0.001; 5 lapses: aOR = 1.17, 95% CI: [1.14, 1.20], *p* < 0.001; 6 lapses: aOR = 1.13, 95% CI: [1.10, 1.16], *p* < 0.001), compared to children with no lapse.

In addition, children seen for non-WCV were associated with higher odds of receiving distal PC versus local PC (aOR = 0.91, 95% CI: [0.90, 0.92], *p* < 0.001), compared to children seen for WCV. Lastly, children in non-rural areas had 74% lower odds of receiving distal PC versus local PC (aOR = 0.26, 95% CI: [0.26, 0.26], *p* < 0.001), compared to children in rural areas. There was no significant association between sex or age and receiving distal PC versus local PC (*p* = 0.095 and *p* = 0.483).

## Discussion

Our study findings contextualize which Medicaid-insured children are at greater risk for experiencing barriers to PC—a critical support for pediatric weight prevention and management [11, 28, 29]. 

### Differences by Race/Ethnicity

Our study found significant differences between racial/ethnic groups and their associations with PC utilization and BMI classification. In this study, Non-Hispanic Black and Hispanic children were more likely than Non-Hispanic White children to be diagnosed as overweight or obese. Conversely, Non-Hispanic Black children, Hispanic children, and children in other racial/ethnic groups are less likely to travel for PC. Similarly, existing literature suggests families with children who identify as a race/ethnicity other than non-Hispanic White may not travel for PC because of disparities in healthcare access, rather than a lack of desire [16, 30]. Equity-oriented interventions that bring healthcare to people in places where they live, study, and work—such as school-based health centers and mobile health clinics—are needed to support PC access and promote comprehensive health across the lifespan for children of all racial and ethnic groups [16, 31–33]. Transportation barriers often restrict access to necessary healthcare services, particularly in rural communities and among children who rely on their family members to attend WCVs [6, 34, 35]. Supportive policy may also facilitate implementation of such interventions to prevent further disparities in healthcare access, due to challenges with Medicaid reimbursement [8, 15]. Nationally, Medicaid expansion policy has increased PC access across racial/ethnic groups to progressively reduce disparities [8, 20]. Previous research found 58 cents per dollar invested in childhood Medicaid is recouped in early adulthood [19]. To date, these policies have been opposed by SC legislators, Non-Hispanic Black individuals in SC would demonstrate the largest coverage gains with Medicaid expansion, which may significantly affect existing disparities between racial/ethnic groups statewide [20]. 

### BMI Classification and Primary Care Utilization

Children diagnosed as overweight or obese are likely to maintain this classification into adulthood, leading to adverse health outcomes [3, 36]. In this study, BMI classification demonstrated significant effects on where children receive PC in SC. Overall, children diagnosed as overweight, relative to healthy weight, have higher odds of receiving distal PC. Further, relative to children with a healthy weight, those diagnosed as obese have higher odds of receiving proximal PC, and lower odds of receiving distal PC than local PC. This finding may be partially explained by factors associated with childhood obesity, including food security, access to healthy food and physical activity opportunities, and socioeconomic status of Medicaid-insured children [8, 11, 20, 37]. Further, this finding is contrary to AAP guidelines, suggesting children diagnosed as obese should receive weekly follow-up visits with their PCP [11]. Particularly among Medicaid-insured children, weekly visits are likely not feasible if traveling for PC [11]. However, community-based interventions, including tailored health promotion programs, may supplement the continuity of care among Medicaid-insured children diagnosed as obese [38–40]. Further research is needed to assess community health needs and facilitate implementation of equitable, evidence-based interventions for adoption of healthy lifestyle behaviors among children.

### Rurality

Finally, rurality had a profound impact on PC utilization. According to this study’s findings, children in rural areas are nearly twice as likely to travel for PC, regardless of the care they need, compared to children in non-rural areas who likely experience much fewer barriers to PC. This places a disproportionate burden on Medicaid-insured children and families in rural communities. In rural SC, Medicaid-insured children have historically been underdiagnosed with overweight and obesity, suggesting that this statistic may underrepresent the vastness of the healthcare gap [41]. Existing research has termed poor longitudinal health outcomes in the southeastern region of the United States, the “southern rural health penalty” [42]. This research largely describes lifestyle behaviors, comorbidities, and associated health outcomes among adults who are overweight and obese; however, pediatric obesity prevention, identification, and treatment is crucial to addressing this geographic “health penalty” [3, 7, 42]. Further research is needed to identify causal relationships for PC utilization and multi-level interventions to optimize equitable healthcare access among pediatric populations [16, 43, 44].

### Need for Comprehensive Obesity Care

Our study findings suggest that Medicaid-insured children who are historically at-risk of poor health outcomes, including Non-Hispanic Black and Hispanic children, children of other racial/ethnic groups, with obesity, and/or who live in rural areas, must also travel for PC. First and foremost, PCPs must understand the role of socioecological factors on childhood obesity, and demonstrate adequate training to improve health literacy and education among patients and their families to prevent and treat pediatric obesity [11, 16, 45]. Existing research suggests that integrated lifestyle behavior counseling and associated support into PC practices may be a successful strategy for assisting PCPs with pediatric weight management and health outcomes [43, 46, 47]. One study in SC found only 3.55% of children received nutrition counseling at wellness visits, and only 12.5% of children were diagnosed as overweight or obese [6]. This study’s findings suggest the reality of children diagnosed as overweight or obese is much higher, describing the reality of the obesity epidemic among Medicaid-insured children in SC. Further, virtual services, including telehealth-based weight management programs, mobile apps, and wearable mobile health technology, have effectively encouraged weight change and adoption of healthy lifestyle behaviors such as nutrition and physical activity across the lifespan [33, 48, 49]. However, Medicaid-insured children may experience additional barriers, including inconsistent insurance coverage for services delivered via telehealth [48, 50]. Further research is needed to understand the impact of implementing virtual interventions across pediatric populations and, specifically, among those with limited PC access.

### Limitations

This study has several limitations. Inherent to this study design, this analysis is limited to examining associations rather than inferring causality between observed variables and distance traveled for PC. Further, a significant proportion of the sample was removed during data cleaning, due to missing race/ethnicity data and a billing processing center in McCormick County that would have skewed the outcomes of this analysis. The use of administrative data also limits contextual factors, including household-level social determinants of health, that may affect one’s likelihood of traveling for PC. Lastly, these findings may be limited in generalizability, due to the unique rural and political landscape of SC related to legislative opposition to Medicaid expansion policies [20]. Still, despite its limitations, this study provides important insights into pediatric PC among Medicaid-insured children in SC and may offer insight to the landscape of healthcare infrastructure across rural and non-rural counties.

## Conclusions

Childhood obesity has evolved to epidemic levels nationwide, requiring a dynamic, multifaceted approach to equitable prevention and weight management. Among Medicaid-insured children in SC, those who are Non-Hispanic White, with diagnosed obesity, with repeated lapses in Medicaid coverage, sought non-WCV, and live in rural counties demonstrate higher odds to travel to a neighboring county for PC, compared to other children. Further, Medicaid-insured children who are Non-Hispanic White, diagnosed as overweight, and have repeated lapses in Medicaid coverage demonstrate higher odds of traveling distally for PC, compared to other children. Further research is needed to understand the causal, complex relationships between patient characteristics, continuous Medicaid coverage, and their effect(s) on obesity. Multi-level policy strategies are needed to promote the adoption of healthy lifestyle behaviors among Medicaid-insured children and to leverage community-based networks, regardless of rurality, for optimal health across the lifespan.

## Data Availability

This data is provided at an aggregate level and is not available for public access following publication. The findings reported are solely that of the research team’s and have not been verified by South Carolina Department of Health and Human Services.
